# A Disease Outbreak in Beef Cattle Associated with *Anaplasma* and *Mycoplasma* Infections

**DOI:** 10.3390/ani13020286

**Published:** 2023-01-13

**Authors:** Karin Persson Waller, Kerstin Dahlgren, Giulio Grandi, Maya Louise Holding, Katarina Näslund, Anna Omazic, Hein Sprong, Karin Ullman, Mikael Leijon

**Affiliations:** 1 Department of Animal Health and Antimicrobial Strategies, National Veterinary Institute (SVA), 75189 Uppsala, Sweden; 2 Vaselunds Gård, Hällestad 381, 24745 Torna Hällestad, Sweden; 3 Department of Microbiology, National Veterinary Institute (SVA), 75189 Uppsala, Sweden; 4 Virology and Pathogenesis Group, UK Health Security Agency, Porton Down, Salisbury SP4 OJG, UK; 5 Health Protection Research Unit in Emerging and Zoonotic Infections, National Institute for Health Research, Liverpool L69 7BE, UK; 6 Department of Chemistry, Environment and Feed Hygiene, National Veterinary Institute (SVA), 75198 Uppsala, Sweden; 7 Centre for Infectious Disease Control, National Institute of Public Health and Environment, 3720 BA Bilthoven, The Netherlands

**Keywords:** beef cattle, whole-genome shotgun sequencing, granulocytic anaplasmosis, *Mycoplasma*, ticks, wild deer, wild boar

## Abstract

**Simple Summary:**

A disease outbreak in a Swedish herd of beef cattle which may be related to a tick-borne infection initiated an in-depth study to investigate the presence of bacteria and viruses in clinically healthy and diseased cattle, as well as in wild deer and boars from the region and in ticks collected from the cattle and deer. The results indicated unusual types of *Mycoplasma* as possible causative factors, probably in combination with immune suppression due to granulocytic anaplasmosis caused by *Anaplasma phagocytophilum*. The latter bacterium was widespread in the herd and was also found in wild deer in the region, and in ticks collected from the cattle and deer. Genetic comparisons indicated that *Anaplasma phagocytophilum* was circulating among the cattle population, while circulation between cattle and deer occurred infrequently.

**Abstract:**

An outbreak of disease in a Swedish beef cattle herd initiated an in-depth study to investigate the presence of bacteria and viruses in the blood of clinically healthy (*n* = 10) and clinically diseased cattle (*n* = 20) using whole-genome shotgun sequencing (WGSS). The occurrence of infectious agents was also investigated in ticks found attached to healthy cattle (*n* = 61) and wild deer (n = 23), and in spleen samples from wild deer (*n* = 30) and wild boars (*n* = 10). Moreover, blood samples from 84 clinically healthy young stock were analysed for antibodies against *Anaplasma phagocytophilum* and *Babesia divergens.* The WGSS revealed the presence of at least three distinct *Mycoplasma* variants that were most closely related to *Mycoplasma wenyonii*. Two of these were very similar to a divergent *M. wenyonii* variant previously only detected in Mexico. These variants tended to be more common in the diseased cattle than in the healthy cattle but were not detected in the ticks or wild animals. The DNA of *A. phagocytophilum* was detected in similar proportions in diseased (33%) and healthy (40%) cattle, while 70% of the deer, 8% of ticks collected from the cattle and 19% of the ticks collected from deer were positive. Almost all the isolates from the cattle, deer and ticks belonged to Ecotype 1. Based on sequencing of the groEL-gene, most isolates of *A. phagocytophilum* from cattle were similar and belonged to a different cluster than the isolates from wild deer. Antibodies against *A. phagocytophilum* were detected in all the analysed samples. In conclusion, uncommon variants of *Mycoplasma* were detected, probably associated with the disease outbreak in combination with immune suppression due to granulocytic anaplasmosis. Moreover, *A. phagocytophilum* was found to be circulating within this cattle population, while circulation between cattle and deer occurred infrequently.

## 1. Introduction

Recently, milder winters with less snow cover, increased precipitation and a prolonged vegetation period, especially in the southern parts of Sweden, have enabled farmers to keep beef cattle (*Bos taurus*) on pasture throughout the year. Milder winters and longer pasture seasons have also improved the survival and abundance of ticks [1]. In Sweden, as in many other European countries, *Ixodes (I.) ricinus* is a tick species commonly associated with the transmission of pathogens to animals and humans [2]. Of such diseases affecting cattle, granulocytic anaplasmosis (GA), caused by infections with *Anaplasma (A.) phagocytophilum*, and babesiosis, in Sweden caused by *Babesia (B.) divergens*, are familiar in veterinary medicine [3,4]. The national incidence of these diseases among Swedish cattle is not known, but in 2019, the incidence of bovine babesiosis was estimated at 6.4% in a questionnaire study [5]. However, the incidence of anaplasmosis or babesiosis may be high in affected herds [4,6,7]. Ticks may also carry other infectious agents that have the potential to cause diseases in animals and humans, e.g., *Borrelia* and *Coxiella burnetti* [2]. Tick-borne diseases in grazing cattle can be difficult to control as wild animals, which may share the pastures, can efficiently support the tick life cycle and act as reservoirs for infections [8,9].

Given the milder conditions mentioned above, ticks can potentially be active throughout the year in some regions, which may change the patterns of tick-borne diseases [1]. In light of this, a farmer in southern Sweden, who keeps beef cattle outdoors all year round, experienced severe illness among some of his calves and young stock, e.g., fever, breathing difficulties, lethargy and/or lameness, starting in early 2020 (i.e., mid-winter). Disease progress was very rapid and several animals died, while some recovered after systemic treatment with oxytetracycline. Based on previous experiences, GA was suspected as a potential cause of the observed symptoms. However, *A. phagocytophilum* DNA was only detected in one of three blood samples sent for analysis. Thus, co-infection or infection with other unknown infectious agents, possibly transmitted by ticks or by some other route of infection, was also considered because GA can cause immune suppression making infected animals more prone to other infectious diseases [10]. Whole-genome shotgun sequencing (WGSS) is a useful tool for the identification of unknown infections in different types of samples without the limitations of traditional microbiological methods, and its use in clinical diagnostics has increased both in human and veterinary medicine [11,12,13]. For the correct interpretation of the results of such analyses, it is important to investigate samples from both diseased and healthy animals.

Therefore, the aim of the present study was to investigate and compare the presence of bacteria, parasites and viruses in the blood from clinically healthy cattle and cattle with clinical disease in this farm by means of WGSS. A further objective was to study the occurrence of infectious agents in ticks found attached to beef cattle from the same farm. Moreover, ticks and spleen samples from wild deer and wild boars from the same area as the farm were also investigated to elucidate the role of wild ungulates as a potential source of tick-borne infections. We hypothesized that the WGSS would detect the presence of other infectious agents than those primarily investigated, which would help in the understanding of the aetiology of the disease outbreak.

## 2. Materials and Methods

### 2.1. Animal and Tick Sampling

All the samples from beef cattle were collected on a beef cattle farm located in the county of Scania in southern Sweden. The cattle on the farm are kept outdoors all year round. From April to July 2020, serum and EDTA-blood were collected by the farm’s veterinarian from 20 animals showing clinical symptoms (e.g., fever, breathing difficulties, lethargy and/or lameness). During this time, blood samples were also taken from clinically healthy cattle. A random selection of samples from 10 of those animals was made for further analyses. Blood samples were also collected twice, in May and September, from 84 clinically healthy young stock. Serum and EDTA-blood were frozen at –20 °C directly after sampling. At the blood sampling of young stock in May and September, approximately 200 ticks attached to the animals were collected and frozen at –20 °C. All the ticks were morphologically identified as *I. ricinus* according to identification keys [14]. Random samples of 32 female *I. ricinus* ticks from May and 29 female *I. ricinus* ticks from September were selected for analyses as described below.

During the hunting season, which ran from October to December (i.e., autumn/early winter), local hunters in the same area as the cattle farm collected the spleens from 15 red deer (*Cervus elaphus*), 11 fallow deer (*Dama dama*), 4 roe deer (*Capreolus capreolus*) and 10 wild boar (*Sus scrofa*) in 2020. The spleens were frozen at –20 °C directly after slaughter. Approximately 50 *I. ricinus* ticks (identified as above) found attached to the wild deer (13 red deer, 8 fallow deer and one roe deer) were collected directly after slaughter and then frozen in sterile test tubes at –20 °C. No ticks were found on the wild boar. A random sample of 35 of these ticks was analysed including at least one tick from each animal with ticks.

### 2.2. DNA and RNA Extraction

Bovine serum, bovine EDTA-blood and spleen samples from wild deer and boar were thawed at room temperature. DNA was extracted from 200 µL EDTA-blood using the ZymoBIOMICS DNA Microprep Kit (Zymo Research, Irvine, CA, USA) and eluted in 20 µL DNase/RNase-free water. RNA was extracted from 200 µL serum using 600 µL TRI Reagent (Sigma-Aldrich, Darmstadt, Germany), purified with Direct-zol RNA Miniprep (Zymo Research, Irvine, CA, USA) and eluted in 50 µL DNase/RNase-free water. Extracted RNA was converted to double-stranded complementary DNA (ds-cDNA) with the SuperScript® IV First-Strand Synthesis System (Thermo Fisher Scientific, Waltham, MA, USA) and Klenow Fragment (New England BioLabs, Ipswich, MA, USA).

DNA extraction of the spleen samples was performed using the EZ1 DNA Tissue Kit (Qiagen, Hilden, Germany). A part of the spleen sample (approximately 40 mg) was cut into pieces and incubated together with 760 µL Buffer G2 and 40 µL Proteinase K at 56 °C for 2–3 hours. After a short centrifugation, 200 µL of the supernatant was extracted using the EZ1 instrument according to the manufacturer´s instructions with an elution volume of 50 µL.

Selected ticks (n = 96) were washed twice in PBS (10%) and left to dry for a few minutes. Each tick was then transferred to a test tube and cut into pieces with a sterile blade. DNA was then extracted using the DNeasy Blood and Tissue kit (Qiagen, Hilden, Germany) according to the manufacturer’s instructions, adding 180 µL ATL buffer and 20 µL Proteinase K in each tube. The digestion step was performed overnight in a 56 °C thermo-block. The extraction proceeded according to the protocol and 50 µL distilled deionized water was used for the final elution.

### 2.3. Control Materials

Synthetic polC gene fragments, 1774 nt long, were obtained (Twist Bioscience, San Franscisco, CA, USA) for *Mycoplasma (M.) wenyonii* str. Massachusetts (GenBank accession CP003703.1) and *M. ovis* str. Michigan (GenBank accession CP006935.1), and a 1773 nt fragment of the polC gene from the INFIAP02-like [15] *M. wenyonii* variant M9473 (Table 1) which was sequenced in the present study (GenBank accession OP852791).

The DNA from samples containing a number of other *Mycoplasma* species, i.e., *M. bovis*, *M. bovigenitalium*, *M. boviculi, M. dispar, M. flocculiare, M. fastidiosum, M. argigini* and *M. haemofeli*s, were obtained from the sample repository of the National Veterinary Institute (SVA), Uppsala, Sweden. A bovine serum sample that was PCR-positive using the Bio-T^®^ *Mycoplasma wenyonii* kit (Biosellal, Dardilly, France) was kindly provided by Prof. Jean-Francois Valarcher (Swedish University of Agricultural Sciences).

### 2.4. Whole Genome Shotgun Sequencing

The concentrations of DNA and ds-cDNA in the samples were measured using a Qubit dsDNA HS Kit (Thermo Fisher Scientific, Waltham, MA, USA) and all the samples were diluted in Tris-HCl (pH 8.5) for use in library preparation with the Nextera XT Library Prep Kit (Illumina, San Diego, CA, USA). The DNA from cattle with a clinical disease was prepared individually while the DNA from the healthy cattle was pooled two by two. Fragmentation of the DNA and the production of libraries with double indices for each sample, including purification with AMPure XP beads (Beckman Coulter, Bra, CA, USA), were performed according to the manufacturer’s instructions and analysed with a High Sensitivity DNA Chip in an Agilent 2100 Bioanalyzer Instrument (Agilent Technologies, Santa Clara, CA, USA). The libraries were pooled and, after denaturation with NaOH and dilution to 12 pM, they were sequenced using the MiSeq reagent kit v3 600 cycles (Illumina, San Diego, CA, USA) on a MiSeq instrument.

In total, 25 DNA samples were sequenced of which 5 contained pools from two healthy animals and 20 samples each came from one animal with a clinical disease. Thus, in total 30 animals were investigated with WGSS (listed in Table 1). In addition, for five of the twenty animals (M7223, M8131, M8458, M9106 and M9473) with a clinical disease, RNA was reverse transcribed and converted to ds-cDNA and subsequently sequenced as described above. 

### 2.5. Bioinformatics

The paired-end reads, on average 2,637,634 per sample, were quality trimmed using a Trimmomatic v 0.39 [16] with a sliding window of four nucleotides and a required average quality score of 15. The trimmed reads were assembled using SPAdes v 3.15.4 [17]. Contigs or quality-trimmed reads were classified using DIAMOND v 2.0.7 [18], which adopts an accelerated blastx [19] method, with a database for classification created using the NCBI nr database and the corresponding NCBI taxonomy databases. Sequences thus classified as potentially relevant microbes with DIAMOND/blastx were classified a second time using blastn [18]. Sequences that kept their taxonomic classification also after blastn analysis were considered valid.

The blood samples from three diseased animals contained large amounts of *Mycoplasma* DNA and allowed the extraction of complete 16S and near-complete polC gene sequences. The polC gene fragments were translated, and the resulting amino acid sequences were aligned with the following selected reference sequences from GenBank: CP006771 (*Mycoplasma parvum,* host: *Sus scrofa*; collected in Indiana, USA, 2013); CP002525 (*Mycoplasma suis*, host: *Sus scrofa*; collected in Illinois, USA); CP040774 (*Mycoplasma bovis*, host: *Odocoileus hemionus*; collected in Nevada, USA, 2012); LWUJ01000013 (*Candidatus Mycoplasma haemobos*, host: *Bos taurus*; collected in Chihuahua, Mexico, 2008); NC_016638 (*Mycoplasma haemocanis*, host: *Canis familiaris*; collected in the USA); CP002808 (*Mycoplasma haemofelis*, host: *Felis catus*; collected in the USA); HE613254 (*Mycoplasma haemominutum*, host: *Felis catus*, collected in Birmingham, UK, 1999); CP003731 (*Candidatus Mycoplasma haemolamae*, host: *Lama pacos*); CP003703 (*Mycoplasma wenyonii*, host: *Bos taurus*; collected in the USA); CP006935 (*Mycoplasma ovis*, host: *Ovis aries*; collected in Michigan, USA, 2012); QKVO01000002 (*Mycoplasma wenyonii*, host: *Bos taurus*; collected in Morelos, Mexico, 2015) and subjected to maximum likelihood phylogeny using CLC Genomics workbench version 21 (Qiagen, Hilden, Germany). Maximum likelihood phylogeny was performed using a neighbourhood-joining starting tree with a general time reversible nucleotide substitution model and a WAG protein substitution model. Bootstrap analysis was performed with 1000 replicates.

### 2.6. Mycoplasma Real Time PCR 

Sequence information revealed the presence of an unusual *Mycoplasma* variant, previously only reported in Mexico [15], in several blood samples from the affected herd. To screen samples for this novel *Mycoplasma*, a specific TaqMan real-time PCR assay targeting the polC gene was designed using the NCBI primer-blast design tool (https://www.ncbi.nlm.nih.gov/tools/primer-blast/ accessed on 4 March 2022). The design was carried out with an explicit specificity requirement towards *Bos taurus* genomes and the Mollicutes class. The primer/probe candidates were further analysed using the CLC Genomics workbench v. 21. The resulting primer and probe sequences were polC12-F: ATTTGAGCTTACCTCCGCCT, polC12-R: GAGGATGTCTTTTCCCGCCTAT and polC12-P: Fam-ACCTTAGAGGAAATTCAAGGCCT-BHQ1. PCR reactions were carried out on a BioRad CFX instrument (Bio-Rad Laboratories, Hercules, CA, USA) utilising the SsoAdvanced Universal Probes Supermix (Bio-Rad Laboratories, Hercules, CA, USA) with the primers and probe at 400 and 200 nM, respectively. The PCR experiments were 40 cycles of 30 s at 60 °C and 10 s at 95 °C following an initial denaturation time of 3 min at 95 °C.

The real-time PCR detection of strain Massachusetts-like *M. wenyonii* was carried out on a BioRad CFX instrument using the Bio-T^®^ *Mycoplasma wenyonii* kit (Biosellal, Dardilly, France) according to the manufacturer’s instructions with the exception that the relative quantification control provided with the kit was used undiluted for the estimation of *Mycoplasma* genome copy numbers (N), assuming an amplification factor of 2 using the formula: N = 10^4^ × 2^Ct,ref−Ct^, where Ct, ref is the Ct-value of the provided quantification control and Ct is the Ct-value of the sample.

### 2.7. Detection of Antibodies to A. phagocytophilum and Babesia divergens

The antibodies to *A. phagocytophilum* were analysed in 84 serum samples collected from 84 young stock in May by an indirect immunofluorescent antibody test according to accredited routines at the National Veterinary Institute, Sweden (SVA4800).

The antibodies to *B. divergens* were analysed in 22 serum samples from 11 young stock by an indirect immunofluorescent antibody test performed in principle as previously described [20]. The antigen was prepared from the blood of a calf infected with *B. divergens,* and FITC-conjugated rabbit anti-bovine IgG (Sigma, code F7887, lot 054M4750V) was used as a secondary antibody at a dilution of 1:400 (as determined by titration against previously used positive and negative control sera). Test sera were diluted 1:40 and 1:160 and only those clearly positive at 1:40 were considered positive.

### 2.8. Detection and Genotyping of A. phagocytophilum

The extracted DNA from bovine EDTA-blood (*n* = 30), spleen samples from wild animals (*n* = 40) and a selection of ticks collected from cattle (*n* = 61) and wild animals (*n* = 35) were analysed using a TaqMan real-time PCR assay targeting the citrate synthase gene (gltA) of *A. phagocytophilum* [21]. The PCR reaction consisted of 10 µL Maxima Probe qPCR Master Mix (Thermo Fisher Scientific, Waltham, MA, USA) and final primer concentrations of 600 nM and a probe concentration of 150 nM, together with RNase-free water and 2 µL DNA in a total volume of 20 µL. The PCR reactions were performed on the CFX96 real-time PCR detection system (Bio-Rad Laboratories, Hercules, CA, USA) using an activation step at 95 °C for 5 min followed by 40 cycles of 95 °C for 15 s and 60 °C for 60 s.

The DNA extracts from samples of bovine EDTA-blood (*n* = 10) and deer spleen (*n* = 19), and from ticks (five from cattle and six from deer) that were positive by a PCR for *A. phagocytophilum* were further analysed at the National Institute of Public Health and Environment in Bilthoven, The Netherlands. Two PCR protocols targeting groEL were used to investigate ecotypes; each sample was investigated using a conventional PCR followed by Sanger sequencing [22]. In short, each reaction of 25 µL consisted of 12.5 µL Hotstart taq Mastermix (Qiagen, Hilden, Germany) and 2 µL of forward and reverse primers at 10 pmol/µL. The PCR programme consisted of 15 min at 95 °C followed by 40 cycles (30 s at 94 °C, 30 s at 57 °C and 45 s at 72 °C) and 10 min at 72 °C. The PCR products were cleaned with ExoSAP-IT™ PCR Product Cleanup Reagent (Applied Biosystems, Foster City, CA, USA) and sequenced using forward and reverse primers by BaseClear (BaseClear, Leiden, The Netherlands). The chromatographs of the sequences were analysed and the primer sites were trimmed in Bionumerics v.7.6 (Applied Maths, Sint-Martens-Latem, Belgium). Ecotypes were assigned to the GroEL sequences as described previously [23].

A recently developed and validated methodology for identifying *A. phagocytophilum* in questing ticks using an ecotype-specific qPCR was also applied to the *A. phagocytophilum*-positive samples [24]. This ecotype-specific qPCR protocol distinguishing between Ecotypes I and II was implemented using IQ-Powermix (Bio-Rad) in a Lightcycler 480 thermal cycler (Roche Diagnostics). Each reaction of 20 µL contained 8 µL DNA extract, 1.6 µL AnaGroEL primer/probe set, 10 µL IQ-Powermix and 0.4 µL water. The program consisted of 5 s at 95 °C followed by 60 cycles (5 s at 94 °C and 35 s at 60 °C) and 10 s at 37 °C. In terms of the methods used, the ecotype-specific qPCR developed seems to be more sensitive than the conventional PCR, whereas the specificity is 100% congruent [24].

### 2.9. Statistical Analyses

The Fisher’s exact test was used to compare the PCR findings of the *Mycoplasma* variants among diseased and healthy cattle. To illustrate these findings, the ratio (fraction of diseased animals infected)/(fraction of healthy animals infected) was also determined. This ratio is infinity for a perfect association with disease, one for no association and zero for a perfect inverse association (i.e., the absence of disease requires the presence of the *Mycoplasma*). This ratio was also calculated for the simultaneous infection of both variants and for uninfected animals. Student’s t-test was used to compare bacterial load in samples with findings of *Mycoplasma* after logarithmic transformation (log10). In addition, the ratio of the geometric means of the concentration of bacteria in blood for diseased and healthy animals was calculated for each *Mycoplasma* variant.

## 3. Results

### 3.1. Analyses of Bovine Blood Samples

**Figure 1:**
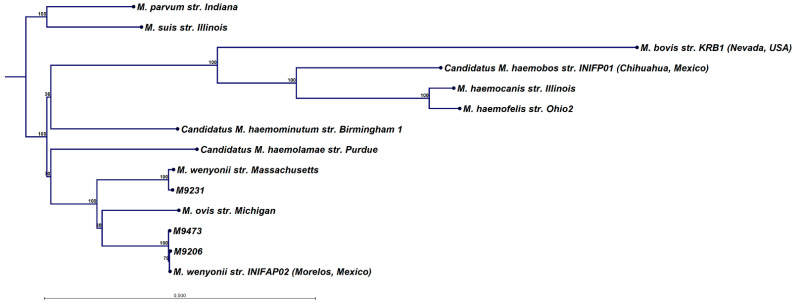
Maximum likelihood phylogenetic tree of the polC polymerase near-complete amino acid sequences (range: 1407-1331 aa) from three samples of the present study (M9231, M9473 and M9206) and various reference samples from GenBank. Percentage bootstrap support of 1000 replicates is shown. The scale bar indicates the average number of substitutions per site.

**Figure 2:**
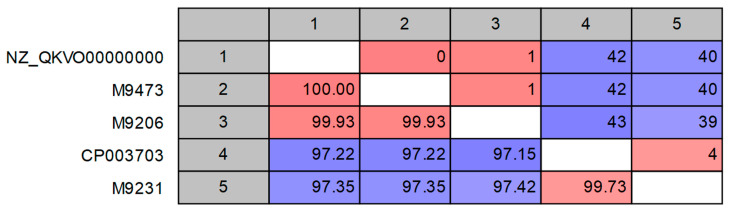
Pairwise comparison of the complete 16S gene of the three *Mycoplasma* isolates M9473, M9206 and M9231 characterised in the present study and the 16S gene of the strains INIFAP02 (NZ_QKVO00000000) and Massachusetts (CP003703). Above the diagonal, the numbers of differences are shown and below the diagonal the percentage identity. Red and blue colour indicate high (<12 differences; >99.5% identity) and low (>24 differences; <98.1% identity) similarity, respectively.

A list of the 30 bovine blood samples collected from the affected herd for which WGSS was carried can be found in Table 1, arranged in collection date order. The main finding from the WGSS data was the presence of the *Mycoplasma* species in the majority of the samples. In particular, three samples, all from diseased animals, gave large numbers of *Mycoplasma* reads and assembly of the sequence reads allowed the determination of the complete polC sequence (samples M9473 and M9206) or a near-complete sequence (sample M9231). These sequences have been deposited at the NCBI GenBank with accessions OP852790-OP852792. Phylogenetic analysis (Figure 1) shows that the two *Mycoplasma* isolates M9473 and M9206 were very similar to a divergent *M. wenyonii* variant previously only found in Mexico, strain INFIAP02 [15], while M9231 was closely related to *M. wenyonii* str. Massachusetts [25]. However, the polC sequences of the two *Mycoplasma* isolates M9473 and M9206, although very similar, were not identical and differed by eight amino acids. In addition, complete 16S genes could be determined for three samples: M9206, M9231 and M9473 (GenBank accessions OP860305-OP860307). Pairwise comparisons between these and the 16S gene of strain INFIAP02 and strain Massachusetts are shown in Figure 2. Again, the M9473 and M9206 sequences were not identical but differed at a single nucleotide, but the 16S gene of M9473 was identical to that of INFIASP02. All three INFIP02-like strains were different at about 40 nucleotides compared with the strain Massachusetts 16S gene, while that of M9231 differed at only four nucleotides (Figure 2). The overall patterns of the 16S gene closely mirrored those of the polC proteins (Figure 1).

Another significant finding from the WGSS data were contigs classified as *A. phagocytophilum* in samples M9336 and M9337. Two contigs of M9336 with lengths of 514 and 430 nt and a single 461 nt contig from sample M9337 were 98%, 99% and 99% identical over the complete contigs lengths, respectively, to the strain Norway variant2 (GenBank accession CP015376) isolated from sheep in Norway in 2010. A third 616 nt contig from M9336 was 99% identical to the strain Norway variant1 (GenBank accession CP046639) over 71% of the contig length (data not shown). The PCR analyses detected *A. phagocytophilum* in 35% of the diseased cattle and in 40% of the healthy cattle (Table 1).

No relevant virus sequences were found in any of the 30 bovine blood samples analysed. There was no evidence of a presence of *Babesia* in the WGSS data.

All 84 serum samples investigated for antibodies against *A. phagocytophilum* were positive. In total, 22 samples were investigated for antibodies to *B. divergens* and none of these samples were positive.

### 3.2. Mycoplasma wenyonii real-time PCR

As mentioned above, three distinct *Mycoplasma* were detected in the herd. An examination of the polC gene of the two INIFAP02-like *Mycoplasma* (M9473 and M9206) showed that no published PCR system was available for the detection of these variants targeting this gene. Likewise, the commercial Biosellal Bio-T kit^®^ was deemed unlikely to detect this species (Biosellal, personal communication) and a real-time PCR assay specific for the INIFAP02-like *M. wenyonii* variants was designed. Dilution of a quantified synthetic gene target of the polC gene showed that the PCR efficiency of this novel assay was in the range of 94–99% and that the analytical sensitivity of the PCR system was about 500 genome equivalents per mL blood (data not shown). The specificity was verified using synthetic gene targets of the *M. wenyonii* strain Massachusetts and *M. ovis* strain Michigan as well as a set of mycoplasmas including *M. bovis, M. bovigenitalium, M. boviculi, M. dispar, M. flocculiare, M. fastidiosum, M. argigini* and *M. haemofelis*. In addition, a bovine serum sample known to be PCR positive for *M. wenyonii* with the Biosellal assay (J.F. Valarcher, personal communication) was tested. The positivity with the Biosellal assay was confirmed with a Ct-value of 24.6. The sample was also positive with the PCR assay developed in the present work with a Ct-value of 36.6.

The results from the two *Mycoplasma* PCR assays are shown in Table 1 and Table 2. Of the 30 bovine samples analysed, 18 (60%) were positive for the *M. wenyonii* type INIFAP02 while 10 (33%) were positive for the *M. wenyonii* type Massachusetts. Both variants were found in a similar range of bacterial loads with 1.5 × 10^1^ – 2.7 × 10^8^ copies/mL and 1.5 × 10^2^ – 3.2 × 10^8^ copies/mL for type INIFAP02 and type Massachusetts, respectively (Table 1).

The results of the Fisher’s exact test for comparisons between diseased and healthy animals were p = 0.14, p = 1.0, p = 1 and p = 0.11 for infection with INIFAP02, with the Massachusetts variant, with both variants and with no variant, respectively. The corresponding ratios comparing diseased and healthy animals were 1.7, 1.1, 1.0 and 0.4, respectively. The bacterial load in the samples positive for *Mycoplasma* (Table 1) did not differ significantly (p = 0.33) between samples with growth of the *Mycoplasma* INIFAP02 or Massachusetts variants. The ratios of the geometric means of the concentration of bacteria in the blood for diseased and healthy animals were 6.0 and 0.9 for the *Mycoplasma* INIFAP02 and Massachusetts variants, respectively. 

### 3.3. Analyses of Spleen Samples from Wild Animals and Ticks from Cattle and Wild Deer

The PCR analyses detected *A. phagocytophilum* in 25–87% of the deer species, with the highest proportion among red deer, but none were detected in the wild boar (Table 2). The *M. wenyonii* INIFAP02-like variant was not detected in any of the samples from the deer or wild boar (Table 2). The samples were not tested for Massachusetts-like variants with the Biosellal assay.

The analyses of ticks originating in cattle found that 8% of these ticks were positive for *A. phagocytophilum* while 17% of the ticks collected from wild deer were positive (Table 2). All the deer, except the roe deer, from which the positive ticks originated, were positive for *A. phagocytophilum* in spleen samples. The *M. wenyonii* strain INIFAP02-like variant was not detected in any of the ticks, while analysis for the Massachusetts-like variant was not performed.

### 3.4. Comparisons of A. phagocytophilum from Cattle, Deer and Ticks

**Figure 3:**
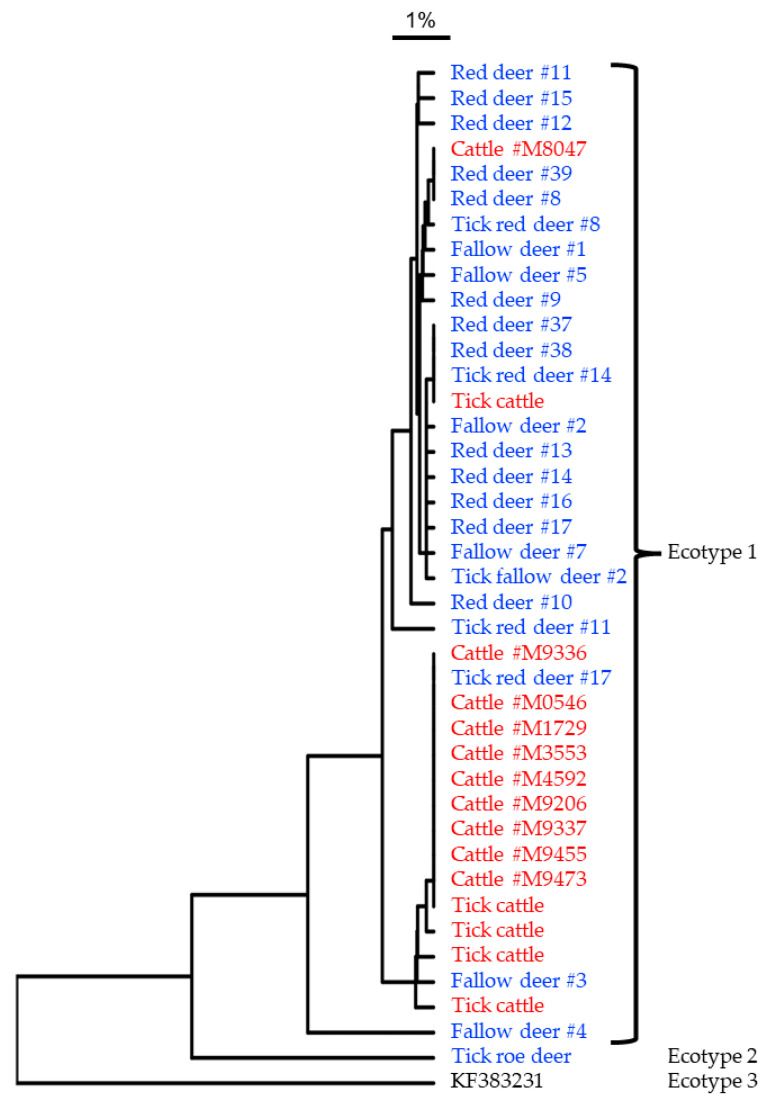
Dendrogram of unweighted pair group method with arithmetic mean (UPGMA) from the multiple sequence alignment of a fragment of the GroEL gene of DNA extracted from blood from cattle, spleen from deer and ticks collected from cattle or deer identified as positive for *Anaplasma phagocytophilum*. Samples and ticks from cattle are written in red and samples and ticks from deer are in blue. Information on *A. phagocytophilum* ecotypes identified by qPCR was included. KF383231 (Ecotype 3) was used as an outgroup.

The genotyping of *A. phagocytophilum* isolates from cattle, fallow deer and red deer found that all isolates belonged to Ecotype 1 (Figure 3). According to the sequencing of the *gro*EL gene, all but one bovine isolate belonged to one genetic cluster within Ecotype 1, while all but one red and fallow deer isolates belonged to other *gro*EL clusters within Ecotype 1. The roe deer sample positive for *A. phagocytophilum* was not available for genotyping. All tick isolates belonged to Ecotype 1, except for one isolate from a roe deer tick that belonged to Ecotype 2. Five pairs of deer and tick isolates were included (e.g., red deer #8 and tick red deer #8) of which four were not identical.

## 4. Discussion

### 4.1. Prevalence of Infectious Agents in Cattle

The finding of two *M. wenyonii*-like variants, INIFAP02-like and Massachusetts-like, was of considerable interest. The importance of these findings for the occurrence of diseases must, however, be interpreted with caution given the small number of animals included in this study. Although the occurrence of the variants was not significantly different between diseased and healthy animals, the ratios estimating the potential associations with diseases for the two *Mycoplasma* variants indicated a possible association between disease and the INIFAP02 variant, but no or a weak association with the Massachusetts variant, but this must be confirmed in further studies. Moreover, the results also indicated that co-infections did not increase the likelihood of disease and that the absence of infection was associated with less disease, but this also needs to be studied further.

Since no PCR assay was available for the detection of INIFAP02-like *M. wenyonii*, a TaqMan PCR assay was developed for this purpose. The specificity in relation to the Massachusetts-like *M. wenyonii* strain was confirmed using a 1773-nt long synthetic fragment encompassing the primer and probe binding sites. In addition, a serum sample positive for *M. wenyonii* with the Biosellal assay (Ct 24.6) was used. In this sample, a low concentration (high Ct-value) of the presumed INIFAP02-like *M. wenyonii* was also found in the specific assay. This could either indicate a slight non-specificity of the developed assay or that this serum sample also contained a small amount of an INIFAP02-like *M. wenyonii*. The serum sample was collected in the county of Uppland about 600 km from the beef cattle farm investigated in this study, which would imply a wide geographical distribution of several divergent variants of *M. wenyonii*. In fact, to the authors’ knowledge, the present study together with the sampling in Uppland (Jean Francois Valarcher, private communication) is the first report of any *M. wenyonii* in Sweden. It should be noted that the number of mismatches between the polC of the original north American *M. wenyonii* strain Massachusetts (accession CP003703) and the PCR assay developed here were 5, 4 and 10 for the forward and reverse primers and probe, respectively, and that this is the same number of mismatches as the *M. wenyonii* strain Massachusetts-like variant sequenced in the present study. An inspection of the bacterial loads presented in Table 1 showed that the sample with by far the largest amount (3.2 × 10^8^ copies/mL blood) of *M. wenyonii* of the Massachusetts variant (M9231) had the lowest amount of INIFAP02-like *M. wenyonii* of all samples that tested positive for this variant (1.5 × 10^1^ copies/mL blood). This indicates that if there was any non-specificity, it was negligible for all samples except M9231, which might be negative for INIFAP02-like *M. wenyonii*. However, if this is the case, it would still not influence any conclusion drawn from the findings of the present study.

Two *M. wenyonii* INIFAP02-like *Mycoplasma*s were found in the present study that differed by eight amino acids in the polC gene and by a single nucleotide in the 16S gene. Thus, it is likely that the *Mycoplasma* circulated for some time in another, unknown reservoir host, diverged, and then contaminated the herd at several time points with different strains or alternatively was maintained in the herd for an extended period of time. Moreover, the mode of transmission is not known, but it has been suggested that insects that can transfer blood may play a role in the spread of *M. wenyonii* [26,27]. To the authors’ knowledge, *M. wenyonii* DNA has only been detected once in a tick in Europe and that was in an *I. ricinus* tick collected from an infected animal [28]. Similarly, the *M. wenyonii* INIFAP02-like strain was not detected in either the ticks or the wild animals in the region in the present study. The similarity with the Mexican strain [15] indicated that this species may represent a new strain with a global spread. *M. wenyonii* is a haemoplasma that infects erythrocytes in cattle. This and another haemoplasma, *M. haemobos*, have been implicated in severe diseases in both Europe and the USA [26,27,29,30]. However, their clinical importance has been questioned because both haemoplasmas have been detected in sick as well as healthy cattle [31,32]. In the present study, the *M. wenyonii* of the strain Massachusetts type was frequently seen in co-infection with the Mexican type. However, the association with disease was most likely non-existent for the Massachusetts type.

As already mentioned, GA was considered as a possible diagnosis at the start of the disease outbreak, and GA is known to occur in cattle during the grazing season in Sweden as well as in several other European countries [4]. Moreover, co-infections of *A. phagocytophilum* and *M. wenyonii* or *M. haemobos* have been reported [29]. In the present study, *A. phagocytophilum* DNA was detected in a substantial but similar proportion of both diseased and healthy cattle. Short contigs of *A. phagocytophilum* infecting the herd were found in two animals and indicated a sequence similarity to variants found to be infecting sheep in Norway in 2010 [33]. These results, together with the fact that all the sampled young stock had seroconverted to *A. phagocytophilum* already in early spring, showed that this infection was very common in the studied herd. Many infected animals are asymptomatic or have mild symptoms that are easily missed. Moreover, it may also be easy to miss an ongoing infection since the amounts of bacteria in the blood vary markedly over time during persistent infections. Thus, the result may be falsely negative. It is also well known that an *A. phagocytophilum* infection often becomes persistent and that such infection can cause immune suppression which leads to an increased risk of other infectious diseases [10].

### 4.2. Importance of Ticks and Wild Animals in the Spread of A. phagocytophilum among Cattle

The findings in this study indicated that a large proportion of the wild deer, but none of the wild boars, was infected with *A. phagocytophilum*. Although the numbers of animals investigated in the present study was too small to establish a prevalence, the result is in line with previous studies from other regions and countries [8,34,35]. To the best of the authors’ knowledge, deer are not clinically affected by the infection. It is also well known that ticks may be infected with *A. phagocytophilum* and spread the bacteria among animals as well as humans [2,3,4]. Thus, it was not surprising to find *A. phagocytophilum* in ticks collected from cattle and deer. 

Previous studies have found different ecotypes of *A. phagocytophilum* in different animal species and ecosystems [23]. In the present study, one isolate from a tick collected from a roe deer was positive for Ecotype 2, while all the other isolates were designated as Ecotype 1. Although an overlap was found between cattle and wildlife genotypes within Ecotype 1, the genotypes from cattle were very similar, if not identical. This suggests that there has been different, but slightly overlapping, transmission cycles of *A. phagocytophilum* Ecotype 1 in cattle and wildlife in the study area.

## 5. Conclusions

To the authors’ knowledge, the present study is the first report of *M. wenyonii* of the strain Massachusetts type in bovine populations in the Nordic countries and the first report of the divergent strain INIFAP02 variant, previously only detected in Mexico, in Europe. Given the small number of animals included in the study, the association between the *Mycoplasma* variants and disease must be interpreted with caution. However, the results indicated strains of an *M. wenyonii* INIFAP02-like species as a possible factor in a disease outbreak in the cattle herd studied, but this needs to be confirmed in further studies. Immune suppression due to infection with *A. phagocytophilum*, which was common in the study herd, could also have been involved. *A. phagocytophilum*, but not the *M. wenyonii* INIFAP02-like strains, was also frequently detected in wild deer in the region as well as in some ticks collected from cattle and wild deer. Genotyping of the isolates indicated that there have been different, but slightly overlapping, transmission cycles of *A. phagocytophilum* Ecotype 1 in cattle and wild deer in the study area.

## Figures and Tables

**Table 1:** Detection of *Anaplasma phagocytophilum* (Ap), *Mycoplasma wenyonii* str INIFAP02-like (MwI) and *M. wenyonii* str Massachusetts-like (MwM) with real-time PCR in blood samples taken from clinically healthy and diseased cattle in spring (April–May) and summer (June–July) 2020.

Animal ID	Day of Sampling	Animal Health Status	Ap (Ct-value)	Quantity (Copies/mL Blood)	
				MwI	MwM
M9276	2020-04-09	Diseased	n.d.^a^	n.d.	n.d.
M9292	2020-04-09	Diseased	37.39	n.d.	n.d.
M8008	2020-04-14	Healthy	n.d.	5.8 × 10^3^	2.4 × 10^6^
M8047	2020-04-14	Healthy	n.d.	7.1 × 10^2^	n.d.
M8119	2020-04-14	Healthy	n.d.	6.5 × 10^4^	6.6 × 10^5^
M8211	2020-04-14	Healthy	n.d.	5.4 × 10^2^	2.7 × 10^3^
M7323	2020-04-14	Diseased	37.15	8.2 × 10^3^	n.d.
M8131	2020-04-14	Diseased	n.d.	3.3 × 10^4^	4.3 × 10^4^
M8180	2020-04-14	Diseased	n.d.	6.1 × 10^3^	1.0 × 10^7^
M8458	2020-04-14	Diseased	n.d.	2.2 × 10^2^	5.4 × 10^3^
M8094	2020-04-14	Diseased	n.d.	1.7 × 10^5^	2.7 × 10^3^
M8249	2020-04-14	Diseased	n.d.	2.8 × 10^2^	3.6 × 10^6^
M9148	2020-05-25	Diseased	n.d.	7.9 × 10^5^	n.d.
M0546	2020-06-02	Healthy	34.42	n.d.	n.d.
M3553	2020-06-02	Healthy	30.20	n.d.	n.d.
M4592	2020-06-02	Healthy	37.32	n.d.	n.d.
M6382	2020-06-02	Healthy	n.d.	n.d.	n.d.
M5911	2020-06-02	Healthy	n.d.	n.d.	n.d.
M7767	2020-06-02	Healthy	n.d.	n.d.	n.d.
M8049	2020-07-08	Diseased	n.d.	3.9 × 10^3^	n.d.
M9206	2020-07-11	Diseased	34.96	2.7 × 10^8^	n.d.
M8279	2020-07-15	Diseased	n.d.	6.3 × 10^4^	n.d.
M9106	2020-07-16	Diseased	n.d.	1.7 × 10^3^	n.d.
M9231	2020-07-16	Diseased	n.d.	1.5 × 10^1^	3.2 × 10^8^
M9336	2020-07-16	Diseased	27.40	n.d.	n.d.
M9337	2020-07-16	Diseased	28.27	4.6 × 10^2^	n.d.
M9455	2020-07-16	Diseased	29.34	n.d.	n.d.
M1729	2020-07-27	Diseased	30.98	n.d.	n.d.
M6259	2020-07-27	Diseased	n.d.	n.d.	1.5 × 10^2^
M9473	2020-07-27	Diseased	36.57	2.5 × 10^8^	n.d.

^a^ n.d. = not detected.

**Table 2:** Summary results of numbers (%) of animals where *Anaplasma phagocytophilum* (Ap+), *Mycoplasma wenyonii* strain INIFAP02-like (MwI+), or *M. wenyonii* strain Massachusetts-like (MwM+) were detected in blood (cattle) or spleen (deer and wild boar) samples and numbers of Ap+ or MwI+ ticks (*Ixodes ricinus*, females) collected from cattle and deer.

Animal Type	Ap+ n/N (%)		MwI+ n/N (%)		MwM+ n/N (%)	
	Animal	Ticks	Animals	Ticks	Animals	Ticks
Cattle (diseased)	7/20 (35)	n.a.^1^	14/20 (70)	n.a.	7/20 (35)	n.a.
Cattle (healthy)	4/10 (40)	n.a.	4/10 (40)	n.a.	3/10 (30)	n.a.
Cattle (healthy)	-^2^	6/61 (8)	-	0/61 (0)	-	-
Fallow deer	7/11 (64)	1/14 (7)	0/11 (0)	0/14 (0)	-	-
Roe deer	1/4 (25)	1/1 (-)	0/4 (0)	0/1 (0)	-	-
Red deer	13/15 (87)	4/22 (18)	0/15 (0)	0/22 (0)	-	-
Wild boar	0/10 (0)	-	0/10 (0)	-	-	-

^1^ n.a. = ticks not available; ^2^ - = samples not analysed.

## Data Availability

The relevant data presented in this study are contained within the article or have been deposited in GenBank (accession numbers given in the results section).
